# Thermally Activated Composite Y_2_O_3_-bTiO_2_ as an Efficient Photocatalyst for Degradation of Azo Dye Reactive Black 5

**DOI:** 10.3390/molecules31010008

**Published:** 2025-12-19

**Authors:** Aleksandar Jovanović, Mladen Bugarčić, Jelena Petrović, Marija Simić, Kristina Žagar Soderžnik, Janez Kovač, Miroslav Sokić

**Affiliations:** 1 Institute for Technology of Nuclear and Other Mineral Raw Materials, Boulevard Franše d’Eperea 86, 11000 Belgrade, Serbia; m.bugarcic@itnms.ac.rs (M.B.); j.petrovic@itnms.ac.rs (J.P.); m.petrovic@itnms.ac.rs (M.S.); m.sokic@itnms.ac.rs (M.S.); 2 Milan Blagojević-Namenska AD, mr Radosa Milovanovica 2A, 32240 Lučani, Serbia; 3 Jožef Stefan Institute, Jamova 39, 1000 Ljubljana, Slovenia; kristina.zagar@ijs.si (K.Ž.S.); janez.kovac@ijs.si (J.K.); 4 Jožef Stefan International Postgraduate School, Jamova Cesta 39, 1000 Ljubljana, Slovenia

**Keywords:** photocatalysis, RB5, water pollution, UV/Vis irradiation, organics decomposition, quantum yield

## Abstract

Water pollution from textile effluents poses serious environmental risks, particularly due to persistent anionic dyes such as Reactive Black 5 (RB5). This study demonstrates that simple deposition of Y_2_O_3_ onto commercially available, biobased TiO_2_ (bTiO_2_) significantly enhances photocatalytic degradation efficiency under simulated sunlight, suppressing rapid recombination of electron–hole pairs. Addressing a key research gap, the proposed method replaces expensive nanoscale precursors and complex synthesis routes typically used for Y_2_O_3_/TiO_2_ systems with a low-cost, straightforward approach involving weak complexation and co-precipitation. The resulting Y_2_O_3_-bTiO_2_ composite was characterized using FTIR, XRD, SEM, EDX, TEM, XPS, and UV-DRS techniques, confirming efficient incorporation of Y_2_O_3_ on the TiO_2_ surface. Photocatalytic experiments revealed that nanoparticles calcined at 700 °C achieved complete RB5 degradation within 60 min—reducing the reaction time by half compared to undoped bTiO_2_. Systematic studies of initial dye concentration, catalyst loading, and irradiation time confirmed that the degradation followed pseudo-first-order kinetics with a rate constant of 0.064 min^−1^ (R^2^ = 0.98). Calculated quantum yields corroborated the reduced electron–hole recombination induced by Y_2_O_3_ deposition. These findings highlight the novelty and practicality of the developed Y_2_O_3_-bTiO_2_ photocatalyst as an efficient, affordable, and environmentally sustainable material for the degradation of industrial dyes.

## 1. Introduction

Water resources are being increasingly contaminated by various organic pollutants originating from textile, pharmaceutical, and agricultural industries [1,2]. Among them, synthetic dyes such as Reactive Black 5 (RB5) are particularly concerning due to their high solubility, chemical stability, and resistance to conventional wastewater treatment methods [3,4]. The persistence of RB5 in aquatic environments leads to significant ecological and health hazards, demanding the development of efficient and sustainable remediation technologies. Advanced oxidation processes (AOPs), especially photocatalysis, have emerged as promising methods capable of degrading such resistant pollutants into environmentally benign products [5,6].

In photocatalytic processes, a semiconductor catalyst is usually illuminated to produce extremely reactive radicals (·OH, O_2_^−^, etc.) that oxidize organic molecules in a non-selective manner. For complex contaminants that are otherwise resistant to biodegradation, such as azo dyes, this method can be quite successful [7]. Because of its high oxidative power, chemical stability, abundance, and low toxicity, titanium dioxide (TiO_2_) is the most commonly utilized photocatalyst in wastewater treatment applications [8]. Additionally, the high availability and prevalence, as well as the affordable cost, have made this a frequently applied oxide. TiO_2_-based catalysts have been used to break down a variety of dangerous substances, including insecticides, dyes, and medications. However, under sun irradiation, pure TiO_2_ performs poorly due to two major constraints. First, TiO_2_ is only photo-active in UV light, which makes up only about 4% of the solar spectrum, due to its wide band gap (~3.2 eV for anatase) [9]. Second, the quantum efficiency of the photocatalytic processes is greatly decreased by the quick recombination of photogenerated electron–hole pairs in TiO_2_ [10]. The quest for changes that can sensitize TiO_2_ to visible light and reduce charge recombination is prompted by these variables, which result in decreased efficiency when employing sunlight or visible-light sources. Several TiO_2_ modification techniques have been studied to address these issues. Important methods for increasing light absorption and fostering charge carrier separation include doping TiO_2_ with metal or non-metal elements and forming heterojunctions by combining TiO_2_ with other semiconductors [11]. By introducing mid-gap energy levels or defect sites, these alterations can allow TiO_2_ to capture visible light and generate additional reactive species.

Rare-earth doping, in particular, has garnered attention as a means of improving TiO_2_ photocatalysis [12,13]. It has been observed that yttrium (Y), one of the rare-earth elements, is a useful dopant for TiO_2_ [14]. By distorting the TiO_2_ lattice and adding oxygen vacancies, Y^3+^ can be incorporated into the lattice (or on its surface), increasing photocatalytic efficiency and extending the range of light absorption [14,15]. The stable yttrium oxide (Y_2_O_3_) has a comparatively modest ionic radius and a band gap of about 6.3 eV [16]. When employed as a co-catalyst or dopant in composite systems, Y_2_O_3_ has been demonstrated to increase the activity of TiO_2_ despite having a wide band gap [17]. By generating surface states and energy levels that promote visible-light excitation, the proper combination of TiO_2_ and Y_2_O_3_ can significantly enhance photocatalytic performance in visible light [14,15]. Jiang et al. created Ag-loaded Y_2_O_3_/TiO_2_ hollow microspheres, which, when exposed to visible light, degraded methyl orange seven times faster than TiO_2_ alone [18]. Similarly, under solar irradiation, various TiO_2_-Y_2_O_3_-based composites, such as those doped with luminous Eu^3+^ and other rare earth co-dopants, have shown better activity than pure TiO_2_ [19,20]. Due to enhanced light absorption, more effective separation, and prolonged maintenance of photo-induced charges at the heterojunction interface, these findings demonstrate that Y_2_O_3_-modified TiO_2_ materials can perform noticeably better than untreated TiO_2_ [15,19]. Despite the fact that many modified photocatalysts have improved performance, there is still a practical problem with the catalyst’s cost and recoverability. Advanced photocatalysts are frequently created as nanoscale particles (such as nano-TiO_2_) or using costly reagents such as titanium alkoxides usually prepared via solvothermal method [12,18,20]. The cumulative expenses associated with catalyst synthesis and operation render AOP technologies economically burdensome for end users. Losses of catalyst material and higher operating costs can result from the requirement for post-treatment filtration or catalyst recovery. For practical water treatment applications, it is therefore much desired to increase the efficiency of commercially accessible (and affordable) TiO_2_ without the need for pricey nanomaterials or laborious recovery procedures.

In order to remove the model dye pollutant Reactive Black 5 (RB5) from water under simulated sunlight, the goal of this study was to create an efficient and reasonably priced photocatalyst. By weakly binding Y^3+^ with glucose on the surface of commercial TiO_2_ and consecutive co-precipitation with ammonium oxalate followed by annealing, we thus created a photocatalytic material using a precursor method. Under solar-simulated light, the photocatalytic activity of this Y_2_O_3_/TiO_2_ composite—which is based on a biobased TiO_2_ powder—was assessed in the breakdown of RB5. The Y_2_O_3_–bTiO_2_ catalyst’s performance was contrasted with data from other TiO_2_-based photocatalysts in the literature and with that of bare TiO_2_. Our goal was to adopt a straightforward deposition technique and steer clear of costly nanoscale additions in order to increase the performance of a commercially available TiO_2_ photocatalyst without appreciably raising total cost. This kind of Y_2_O_3_-modified TiO_2_ composite (made using a weak complexation and co-precipitation method) has not been documented before, according to the literature review. The encouraging outcomes of this study implies that a Y_2_O_3_-bTiO_2_ catalyst of this kind may be a viable option for efficiently and economically treating wastewater streams that contain dyes or even other organic contaminants.

## 2. Results

### 2.1. Structural Characterization

#### 2.1.1. FTIR and XRD

**Figure 1:**
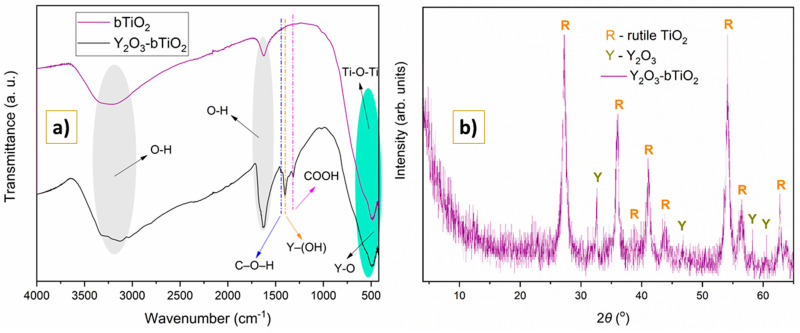
FTIR spectra of bTiO_2_ and Y_2_O_3_-bTiO_2_ particles (**a**); XRD of Y_2_O_3_-bTiO_2_ particles (**b**).

Figure 1a presents IR spectrums of base and modified photocatalytic materials.

Three autonomous IR regions were identified in the bTiO_2_ nanoparticles’ absorption peaks (purple line in Figure 1): 480–515; 1629; and 3159–3382 cm^−1^. The stretching vibrations of the O-H bond have been observed as the cause of the wide, broad peak at 3344–3144 cm^−1^ [19]. Additionally, the bending of the −OH group was linked to the peak at 1650 cm^−1^. In the rutile-phased bTiO_2_ crystal structure [1], the bending of Ti-O-Ti bands is responsible for the vibrational energy detected in the 500–600 cm^−1^ region [21].

After depositing Y_2_O_3_, several changes in the IR spectrum of synthesized Y_2_O_3_-bTiO_2_ (black line in Figure 1) were observed. Firstly, the novel vibrations of the C–O–H groups were observed at 1443 cm^−1^ [22]. Secondly, the intensive, sharp peak located at 1390 cm^−1^ may be associated with the Y–(OH) bond [23,24]. In addition, the symmetrical and asymmetrical vibrations of the COOH are indicated by the non-intensive peak at 1319 cm^−1^ [25]. Also, the peak at 1650 cm^−1^ detected in both materials was intensified in the modified photocatalyst, possible due to enlarged surface vacancies in fabricated composite. Lastly, Ti-O and Y-O bonding contributed to the wide peak found in the region of 500–550 cm^−1^ [26].

Figure 1b presents the XRD spectrum of fabricated composite. Intensive peaks appeared at 2*θ* of 27.17°, 36.01°, 38.62°, 40.99°, 43.79°, 53.99°, 56.46°, and 62.75°, indicating the rutile-phase nature of base material bTiO_2_ [1]. After the synthesis of photocatalyst Y_2_O_3_-bTiO_2_, new peaks at 2*θ* of 32.78°, 46.87°, 58.21°, and 60.47° were observed, which correspond to Y_2_O_3_ [15,27].

The above-mentioned facts (peaks observed in Figure 1a,b) truly indicated that the Y_2_O_3_ nanoparticles were deposited effectively on the bTiO_2_ surface, proving that the Y_2_O_3_-bTiO_2_ nanocomposite had formed successfully.

#### 2.1.2. SEM and EDX

**Figure 2:**
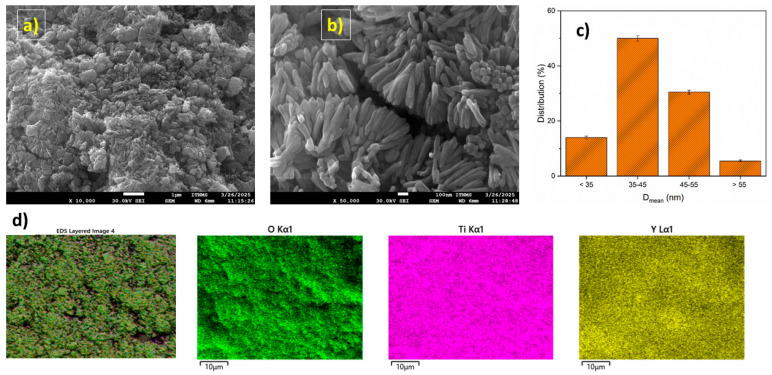
SEM scans of Y_2_O_3_-bTiO_2_ particles at 10,000× (**a**) and 50,000× (**b**) magnification; particle diameter distribution (**c**) and EDS scan of Y_2_O_3_-bTiO_2_ surface (**d**).

Figure 2 presents SEM scans of the modified photocatalyst at magnifications of 10,000 (Figure 2a) and 50,000 (Figure 2b) times. From Figure 2a,b, it can be observed that the fabricated particles are rod-shaped. According to SEM analysis, particle dimensions were mostly in the range of 35–45 nm. The distribution is mostly uniform (Figure 2c). However, there were slight clusters between the particles because the diameters of the particles were slightly reduced after recombination, and the nanoparticles with small diameters were more likely to agglomerate. Results demonstrated that the further enlargement of bTiO_2_ particles was suppressed by the inclusion of Y_2_O_3_ in the proper proportion.

To determine the chemical composition of Y_2_O_3_-bTiO_2_, mapping of the photocatalyst surface was carried out. EDX (Figure 2d) analysis proves the presence of the following elements in descending order: Ti (49.8%), O (36.2%), and Y (8.5%). Other elements like Al, Si, and Cl were detected in sum under 5%, which represent impurities probably in used reactants during synthesis. The successful deposition of Y_2_O_3_ over TiO_2_ and its consistent distribution throughout the surface were demonstrated. This could be crucial for the photocatalytic activity of fabricated composite in the presence of lamp irradiation.

#### 2.1.3. TEM

**Figure 3:**
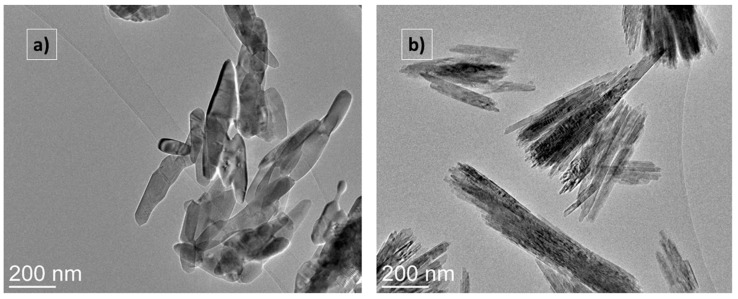
Bright-field TEM images of bTiO_2_ (**a**) and Y_2_O_3_-bTiO_2_ (**b**) particles.

Figure 3 shows the TEM scans of the base bTiO_2_ (a) and chemically produced Y_2_O_3_-bTiO_2_ (b) nanoparticles.

Figure 3 shows bTiO_2_ (a) and Y_2_O_3_-bTiO_2_ (b) particles. Y_2_O_3_-bTiO_2_ (b) particles have a tendency to coalesce into clusters that range in size from 200 nm to 600 nm. The particles maintain their original shape and mostly mimic pure bTiO_2_ particles. Only changes in the sharpness and thickness of formed agglomerates are detected. Furthermore, the composite samples’ extremely thin Y_2_O_3_ coating layer corresponds with the EDX analysis (Figure 2) and the absence of intensive IR peaks associated with the deposition of Y_2_O_3_ particles onto bTiO_2_ in Figure 1.

#### 2.1.4. XPS

In order to gain insight into the surface chemistry, we performed XPS analyses of surface composition and oxidation states of elements. We found the following elements on the surface: Ti, O, and C. On the sample Y_2_O_3_-bTiO_2_, we also found Y and Cl. Surface composition in at.% is given in Table 1.

As presented in Table 1, the Y_2_O_3_–bTiO_2_ composite exhibits a significantly different chemical composition compared to the parent bTiO_2_ material. These differences arise primarily from the distinct synthesis procedures used for the two samples. The initial bTiO_2_ was prepared from titanium isopropoxide and mandarin peel extract without any subsequent high-temperature treatment. Consequently, organic compounds originating from the natural extract remained embedded in the material, as confirmed by the elevated carbon content detected in bTiO_2_. The presence of carbon in the TiO_2_-based photocatalyst can play a crucial role and positively affect the efficiency of the photocatalytic reaction by enhancing stability of the synthesized material, boosting UV uptake, and advancing charge separation [28,29,30]. Moreover, carbon present in TiO_2_-based photocatalysts has been reported to introduce intraband-gap states near the valence band, which can modify light absorption behavior and influence charge carrier dynamics, while carbonate and elemental carbon species on the surface may additionally enhance adsorption of organic molecules and stabilize reactive intermediates during photocatalysis [31,32,33]. In contrast, the Y_2_O_3_–bTiO_2_ composite was synthesized through a three-step process involving weak binding, co-precipitation, and calcination. During calcination, residual organic components were oxidized and removed as gaseous products, while yttrium oxide was simultaneously formed and anchored onto the TiO_2_ surface. Interestingly, the surface carbon content in the calcined composite increased slightly, which is consistent with the findings reported by Mikołajczyk et al. [12]. In their work, rare-earth-modified TiO_2_ prepared by a solvothermal route also exhibited surface enrichment with carbon-containing species, attributed to the adsorption of CO_2_ from the atmosphere onto basic surface sites. A similar phenomenon likely occurred in the present study, where CO_2_ molecules became physically adsorbed on the surface of Y_2_O_3_–bTiO_2_, leading to a comparable carbon content despite the high-temperature treatment. These results were not obtained by the SEM-EDX analysis, most likely due to a different analyses depth, which is much larger for EDX and what makes EDX more bulk-sensitive. The XPS technique is much more surface-sensitive (5 nm), which makes the detection of carbon-based surface species easier.

It is also important to note that this analytical method detected relatively higher chlorine content compared to the results obtained by SEM-EDX. However, such discrepancies are expected, as the recent literature reports have emphasized the uncertainty of this technique for elements present in trace amounts [34].

**Figure 4:**
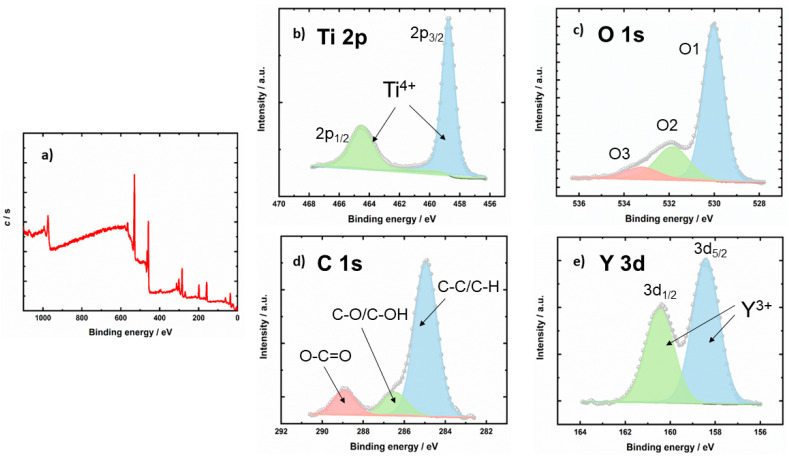
Wide-energy-range XPS spectrum of Y_2_O_3_-bTiO_2_ sample (**a**); deconvolution of Ti 2p spectrum from Y_2_O_3_-bTiO_2_ sample (**b**); deconvolution of O 1s spectrum from Y_2_O_3_-bTiO_2_ sample (**c**); deconvolution of C 1s spectrum from Y_2_O_3_-bTiO_2_ sample (**d**); deconvolution of Y 3d spectrum from Y_2_O_3_-bTiO_2_ sample (**e**).

High-energy resolution XPS spectra Ti 2p, O 1s, C 1s, and Y 3d were acquired, and spectra were deconvoluted. Deconvoluted XPS spectra are shown in Figure 4.

Figure 4b shows the Ti 2p spectrum, which is composed of Ti 2p_3/2_ and 2p_1/2_ peaks. The Ti 2p_3/2_ peak is at 458.7 eV. This binding energy shows the Ti^4+^ oxidation state in the bTiO_2_ lattice [35]. Figure 4c shows the O 1s spectrum, which is composed of three peaks named O1, O2, and O3. O1 at 530.0 eV is from O^2-^ in the bTiO_2_ oxide lattice; O2’s peak at 531.9 eV may be due to oxygen vacancies in the bTiO_2_ lattice or OH groups. The O3 peak at 533.2 may be due to O-H/H_2_O bonds [36]. Figure 4d shows the C 1s spectrum, which comprises three peaks. The first peak at 284.9 eV is assigned to C-C/C-H bonds (due to residual organic component in bTiO_2_), the second peak at 286.5 eV may be due to C-O/C-OH bonds, while the third peak at 288.9 eV may be assigned to O-C=O [37]. Figure 4e shows the Y 3d spectrum, which is composed of Y 3d_5/2_ and 3d_3/2_ peaks separated by 2.05 eV. By deconvolution of this spectrum, we found that the Y 3d_5/2_ peak is at 158.4 eV. This binding energy shows the Y^3+^ oxidation state [38].

#### 2.1.5. UV-DRS

**Figure 5:**
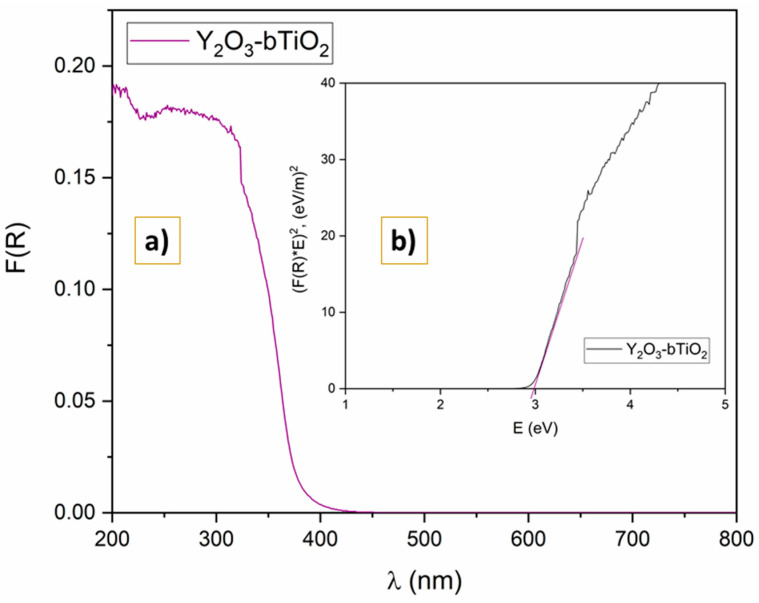
Optical activity of the sample Y_2_O_3_-bTiO_2_ (**a**); Tauc plot of Y_2_O_3_-bTiO_2_ specimen (**b**).

The band gap energy of Y_2_O_3_-bTiO_2_ composite particles was determined using optical absorption spectra recorded by a UV spectroscopy. In Figure 5 are shown the collected UV-Vis spectra of the Y_2_O_3_-bTiO_2_ specimen. The visible area exhibits a modest shift towards the longer wavelength compared to commercial TiO_2_ P25 [39].

The inset graph (Figure 5b) demonstrates a Tauc graph for fabricated composite. A bandgap energy of 2.99 eV has been identified explicitly, indicating a lowering band gap compared to base bTiO_2_ material [1]. Also, fabricated composite has a band gap lower than commercial TiO_2_ P25 (3.3 eV), revealing the potential for improved photocatalytic properties [40]. The band gap may narrow as a consequence of the localized electronic states induced by the inherent flaws between the valence and conduction layers of the TiO_2_ molecule. As a result, electrons migrate easily from valence to conduction bands due to the smaller barrier. Therefore, they are boosting photocatalytic efficiency toward the model pollutant.

### 2.2. Photocatalytic Degradation

After thermal activation of prepared nanocomposite at 700 °C, the obtained photocatalyst was employed in the photocatalytic assay.

**Figure 6:**
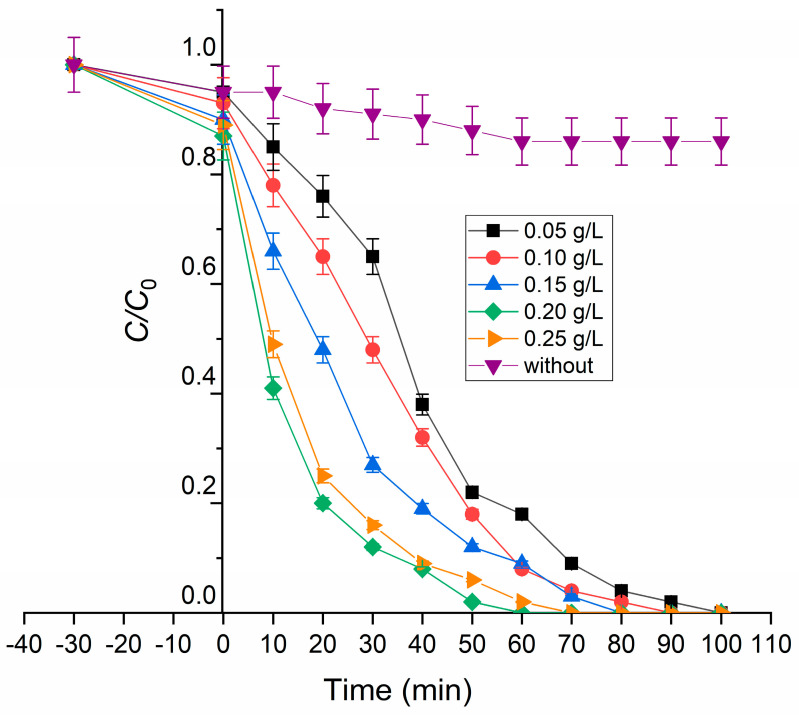
Influence of catalyst dose on process efficiency.

Figure 6 gives degradation curves by varying catalyst amount (0.05–0.25 g/L), under proposed operational conditions for 100 min. In the first 30 min (left part, from t = 0 min, of curves on Figure 6), the reaction was executed without irradiation with the aim of achieving adsorption equilibrium on the catalyst surface. Observed reduction in RB5 concentration was in range from 5 to 10% by increasing the initial catalyst amount. A similar trend is already shown in the available literature [41,42].

As a measure of persistence of RB5 dye, a photolysis test was performed. After 120 min, insufficient degradation was observed (purple line on Figure 6). The degradation rate was only 14% without setting the pH value of the initial dye solution; thus, photolysis was not investigated further.

As can be seen from Figure 6, the best results were in the system with a catalyst dosage of 0.20 g/L. The photocatalytic degradation rate increases with rising catalyst dosage because a greater number of active sites become available for photon absorption and pollutant oxidation [43,44]. However, when the photocatalyst concentration exceeds an optimal level, excessive particle aggregation and light scattering occur, reducing the effective irradiation area and leading to a decline in degradation efficiency [45,46]. A lower amount of the catalyst in the same reaction solution but higher than 0.20 g/L gives inferior results. After 60 min of irradiation, the decolorization efficiencies were 99% (0.20 g/L), 98% (0.25 g/L), 92% (0.15 g/L), 91% (0.10 g/L), and 82% (0.05 g/L).

**Figure 7:**
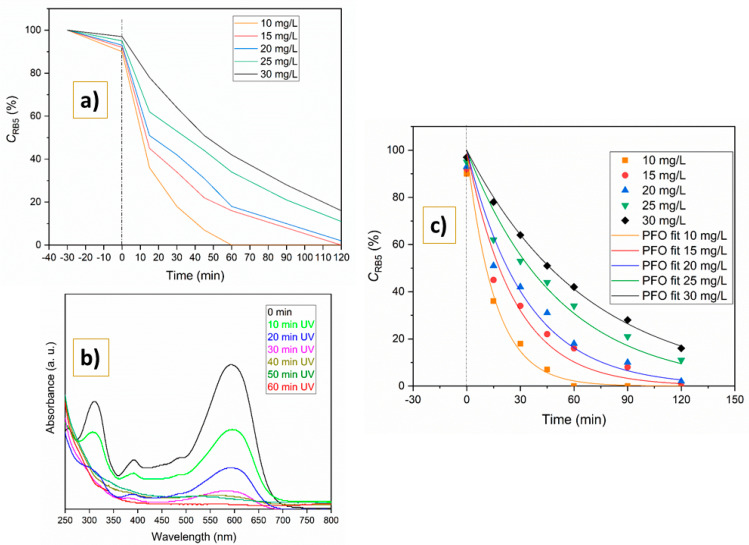
Influence of initial RB5 concentration on process efficiency during time (**a**), and UV spectra using 10 mg/L of dye and 0.20 g/L of Y_2_O_3_-bTiO_2_ photocatalyst (**b**); fitting of experimental data with PFO rate equation (**c**).

When the influence of the initial RB5 concentration was examined (Figure 7), notable variations in degradation efficiency were observed. The initial dye concentration varied between 10 and 30 mg/L, while the photocatalyst amount was kept at 0.20 g/L. Considering a starting concentration of 10 mg/L throughout the first 60 min of irradiation, the degradation rate of RB5 was almost 100%, as illustrated in Figure 7. With each 5 mg/L increase in pollutant concentration, the degradation efficiency decreased continually from 99.9% at 10 mg/L to 84.1%, 72.3%, 66.4%, and 57.9% at 15, 20, 25, and 30 mg/L, respectively.

Our findings and earlier research on Y_2_O_3_-modified TiO_2_ systems suggest that enhanced charge separation at the Y_2_O_3_/TiO_2_ interface is the most likely photocatalytic mechanism. It is anticipated that Y_2_O_3_ will increase the availability of photogenerated charge carriers for surface reactions by promoting interfacial electron transfer and inhibiting recombination. Consequently, the Y_2_O_3_–bTiO_2_ composite exhibits a faster degradation of RB5 due to the increased production of reactive oxygen species such hydroxyl and superoxide radicals.

Our findings and related studies on Y_2_O_3_-modified TiO_2_ systems indicate that the improved photocatalytic activity primarily originates from suppressed electron–hole recombination at the Y_2_O_3_/TiO_2_ interface [15,20,27]. The incorporation of Y_2_O_3_ alters the surface electronic environment of TiO_2_, enabling photogenerated electrons to be efficiently trapped at interfacial sites associated with Y^3+^ species [12,47]. This localized excess negative charge stabilizes photoinduced electrons and prevents their rapid recombination with holes, thereby prolonging the lifetime of photogenerated charge carriers under irradiation.

As a consequence of reduced recombination, photogenerated holes remain available for a longer period to participate in oxidation reactions at the catalyst surface. These holes react readily with adsorbed water molecules and surface hydroxyl groups, promoting the formation of hydroxyl radicals that are highly effective in degrading RB5. At the same time, stabilized electrons can react with dissolved oxygen to generate superoxide radicals. The combined increase in the availability and lifetime of reactive charge carriers, driven by inhibited recombination rather than improved separation, leads to enhanced reactive oxygen species production and explains the accelerated photocatalytic degradation observed for the Y_2_O_3_–bTiO_2_ composite.

It is evident from Figure 7b that RB5 exhibited two distinctive absorption peaks at 310 and 593 nm. A decline in absorbance values was noted over the reaction period. There were no additional peaks that emerged throughout the entire illumination procedure, suggesting that RB5 was effectively broken down.

Collected results presented in Figure 7a were fitted using the Langmuir–Hinshelwood Equation (Equation (1)) to achieve agreement with pseudo-first-order law. The graphical representation of the results is shown in Figure 7c, while the calculated rate constants (*k*) and half-reaction times (*t*_0.5_) are summarized in Table 2.

High correlation coefficients (R^2^ ≥ 0.95) were obtained for all tested initial dye concentrations, confirming an excellent fit to the kinetic model of pseudo-first-order (PFO) law. As the RB5 concentration increased, the rate constant (*k*) decreased more than fourfold (from 0.064 to 0.015 min^−1^), mainly due to the competition for active sites on the photocatalyst surface and partially due to reduced light penetration through the solution [48].

### 2.3. Quantum Yield and Multicycle Degradation

The gradual decline in photocatalytic efficiency over successive cycles can be attributed mainly to changes in the physical and surface properties of the Y_2_O_3_–bTiO_2_ composite rather than to chemical instability. During repeated use and drying, the catalyst particles may undergo partial aggregation, leading to a reduction in the specific surface area and decreased accessibility of light to the photocatalytically active sites [49,50]. Additionally, surface adsorption of dye molecules and reaction intermediates can alter the surface charge and zeta potential, weakening the electrostatic interaction between the catalyst and the anionic RB5 molecules and thereby slowing down the degradation kinetics.

Although the catalyst maintained structural integrity and no yttrium leaching was detected in the rinsing solution, prolonged irradiation could have induced minor structural or textural modifications such as decreased surface roughness or the formation of recombination centers [51]. These defects may facilitate electron–hole recombination, thereby lowering the generation rate of reactive oxygen species responsible for pollutant degradation [52,53]. Nevertheless, maintaining more than 80% of the initial efficiency after five cycles demonstrates excellent stability and reusability of the Y_2_O_3_–bTiO_2_ photocatalyst, confirming its suitability for practical and sustainable wastewater treatment applications.

**Figure 8:**
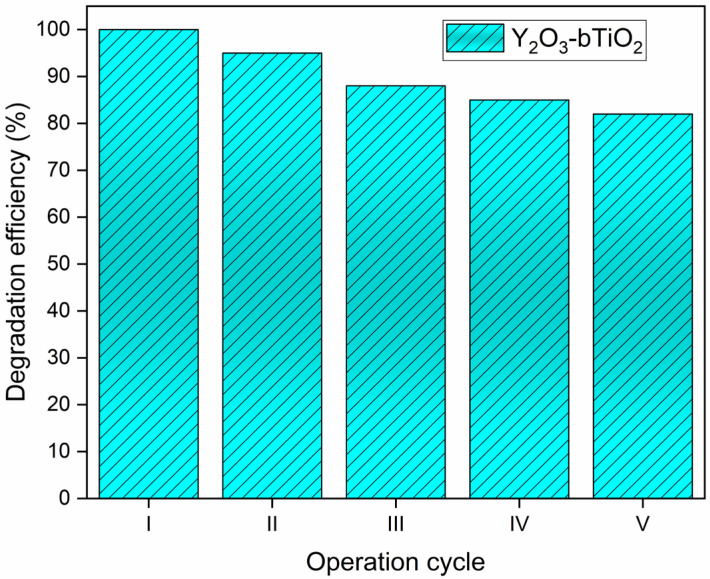
Testing of photocatalyst reusability test for the removal of RB5 within five consecutive operational reactions (catalyst dosage= 0.20 g/L, C_0_(RB5) = 10 mg/L).

Strong linkages among the bTiO_2_ carrier and the Y_2_O_3_ precipitate strengthen endurance, as evidenced (Figure 8) by the employed photocatalysts’ degradation rate decreasing significantly from 100% to 90% after the third reaction cycle.

Together with UV/Vis measurements of residual concentrations of RB5 during photocatalysis, values of quantum yield were determined. The calculated quantum yields (*Φ*) for photocatalytic assays of RB5 degradation are given in Table 2.

Determination of *Φ* values is one of the best auxiliary parameters in photochemistry. Namely, it gives better insights into the number of absorbed photons by reaction solution during irradiation, per time unit. The fluctuation of obtained *Φ* values (Table 3) has a similar trend (directly proportional) to photocatalytic efficiency (Table 2). Quantum yields of 0.61 (at reaction with 10 mg/L of RB5) and 0.11 (at reaction with 30 mg/L of RB5) obtained for Y_2_O_3_-bTiO_2_ show that the fabricated photocatalyst has the ability to generate oxidative species in the reaction suspension under the influence of a sun-imitated lamp source. Therefore, results from the quantum yield assay prove the photocatalytic efficiency of the synthesized Y_2_O_3_-bTiO_2_ composite.

## 3. Discussion

The degradation of RB5 under simulated sunlight was employed to evaluate the photocatalytic performance of the synthesized Y_2_O_3_–bTiO_2_ composite. Prior to photocatalytic testing, the structural and textural characteristics of the catalyst were analyzed.

FTIR spectra confirmed the presence of characteristic Ti–O and Ti–O–Ti vibrations in both bTiO_2_ and Y_2_O_3_–bTiO_2_, while Y–OH vibrations appeared exclusively in the Y_2_O_3_–bTiO_2_ sample. The Y–O–Ti bonds were likely overlapped by the dominant Ti–O–Ti vibrations in the 500–700 cm^−1^ range, preventing their distinct identification. The XRD diffractogram proves the presence of two mineralogical phases in the fabricated photocatalyst, Y_2_O_3_ and TiO_2_. SEM analysis revealed the formation of compact particle clusters after thermal activation at 700 °C, reflecting the particles’ tendency to minimize surface energy and slightly reduce the specific surface area. Accompanying EDS analysis shows uniform distribution of main elements (Ti, O in bTiO_2_ and Ti, O, and Y in Y_2_O_3_–bTiO_2_), proving successful obtaining the proposed Y_2_O_3_–bTiO_2_ photocatalyst. Simultaneously, the high-temperature calcination promoted partial crystallization and enhanced interfacial bonding between TiO_2_ and Y_2_O_3_ phases. This process yielded sharper and more defined particle edges, suggesting improved crystallinity and lattice ordering [54,55]. These morphological changes are consistent with thermally induced crystallization phenomena reported in related studies and are expected to contribute to improved charge transport and photocatalytic stability. XPS analysis further confirmed the successful deposition of Y_2_O_3_ on the bTiO_2_ surface, as evidenced by the Y 3d_5/2_ and Y 3d_3/2_ peaks at 158.4 eV and 160.4 eV, respectively [38].

Photocatalytic assay has shown the complexity of the degradation of organic molecules under the influence of UV radiation. The first operational parameter that was investigated was catalyst amount (0.05–0.25 g/L), for 100 min of irradiation, by fixing initial RB5 concentration at 10 mg/L. The reaction part performed in dark (first 30 min) shows that pure adsorption part is not the main route of RB5 removal form observed reaction system, since the observed decrease was maximally 10% for a catalyst amount of 0.25 g/L. Upon addition of UV light, the best photocatalytic efficiency (99%) was observed when the reaction suspension consisted of 0.20 g/L of Y_2_O_3_-bTiO_2_ and 10 mg/L of RB5. Lower catalyst loadings (0.05–0.15 g/L) resulted in reduced degradation, primarily due to the smaller number of active sites available for photon absorption and e^−^/h^+^ pair generation. Conversely, a higher concentration of the photocatalyst (0.25 g/L) gives similar results. Noticed trends correlate with the possibility of sunlight breakthrough solution. Namely, higher concentrations of the photocatalyst lead to increased turbidity of the reaction suspension and possible coagulation of added photocatalyst. This phenomenon leads to the blockage of active sites on the photocatalyst surface, reducing the numbers of generated pairs e^−^/h^+^. Other authors confirm a similar trend [56,57,58,59,60].

The second investigated parameter was initial RB5 concentration. The best results were seen in the system with an initial dye concentration of 10 mg/L and 0.20 g/L of catalyst, within 90 min. The lowest concentration gives the best results, while increasing the initial concentration follows a decrease in photocatalytic efficiency, from 99.9 to 57.9%, which is followed by values of rate constants *k*. This trend is attributed to excessive dye adsorption on the catalyst surface, which inhibits the generation of reactive oxygen species by restricting the access of photons to the active sites. Also, in that way, an exaggerated dose of dye molecules covers active sites on the surface and suppresses the generation of reactive species (e.g., OH^−^ radicals) involved in organics decomposition [48,61,62].

The kinetic data fit well with the pseudo-first-order model (Equation (1)), yielding high correlation coefficients (R^2^ > 0.98). For the optimal system (0.20 g/L catalyst, 10 mg/L RB5), the rate constant (*k*) was 0.064 min^−1^, with a corresponding half-life (t_0.5_) of 10.9 min, confirming the rapid degradation kinetics.

The auxiliary parameter that was determined was quantum yield (*Φ*) of photodegradation reaction. Obtained values are in agreement with determined catalyst efficiency at different concentrations of dye and calculated values of rate constants. *Φ* represents the potency of the photooxidative system as a measure of generated photons in the observed volume of the system. Received photons from the UV lamp are necessary for excitation on the catalyst surface and production of pairs e^−^/h^+^, which lead into generation of OH^−^ radicals responsible for the decomposition of the pollutant.

The final step in the assessment of the Y_2_O_3_–bTiO_2_ photocatalyst involved its reuse in consecutive photodegradation cycles, where the material demonstrated good structural stability and sustained photoactivity over five successive runs, with only a moderate decrease in degradation efficiency to about 85%.

The following Table 4 gives similar photocatalytical systems for the degradation of various pollutants.

As can be seen from Table 4, different TiO_2_-based photocatalysts are mostly used in photocatalytic degradation of dyes like methyl orange and Reactive Black 5. Mikołajczyk et al. [12] used Y-TiO_2_ for the degradation of phenol. Low degradation efficiency (14%) confirms the need for further development of photocatalytic materials for the degradation of phenolic compounds. In studies where methyl orange was the model pollutant, enhanced efficiencies (>80%) were obtained using Y-TiO_2_-H_2_ [14], Ag-TiO_2_/Y_2_O_3_ [18], and Y_2_O_3_/TiO_2_-Y_2_TiO_5_/CNT [20]. To the best of our knowledge, photodegradation of Reactive Black 5 aqueous solution, except in our study, was performed in only one paper using Y/TiO_2_-based photocatalysts. In a paper by Ren et al. [15], the reactive solution was decolorized with Y_2_O_3_/TiO_2_-Loaded Polyester Fabric. After 150 min of reaction, observed decolorization of the dye solution was 83%, with k = 0.47846 min^−1^. In our study, employing biobased Y_2_O_3_-bTiO_2_ gives nearly complete degradation of RB5 solution with a process efficiency of 99%. Only the photocatalyst Y-TiO_2_-H_2_ synthesized in the study of Li et al. [14] achieved better results with a slightly shorter reaction time and a smaller mass of catalyst used. Compared to other available studies in the literature, our system gives prominent results toward photodegradation of Reactive Black 5 dye.

The superior photocatalytic performance of the composite photocatalyst can be attributed to the synergistic interaction between the Y_2_O_3_ and TiO_2_ phases, which suppresses charge carrier recombination and thus prolongs the lifetime of photogenerated electron–hole pairs [12,15]. The incorporation of Y_2_O_3_ likely introduces shallow trap states near the conduction band of TiO_2_, facilitating efficient electron transfer and reducing recombination losses. Moreover, the increased crystallinity and improved interfacial contact formed during calcination contribute to more efficient charge transport and surface reaction kinetics [63]. The presence of Y^3+^ ions also enhances surface basicity, promoting greater adsorption of anionic dye molecules such as RB5, which further supports higher degradation efficiency [64,65]. These findings confirm that the developed photocatalyst not only provides a cost-effective alternative to nanostructured TiO_2_-based materials but also exhibits strong photostability and reusability, positioning Y_2_O_3_–bTiO_2_ as a viable and sustainable candidate for large-scale wastewater treatment applications [66].

## 4. Materials and Methods

### 4.1. Fabrication of Y_2_O_3_-bTiO_2_

Synthesis of nanocomposite particles consisted of two major steps: obtaining bTiO_2_ particles from mandarin peel extract and further modification with Y_2_O_3_ particles.

#### 4.1.1. Obtaining bTiO_2_ Particles

Details of a procedure for greener obtaining of biobased titanium dioxide (bTiO_2_) particles are described previously in a paper by Jovanovic et al. [1]. Shortly, after washing and grinding mandarin peels, obtained parts were then added to a flask filled with warm water at 60 °C for 120 min. Produced extract was filtered and placed in a three-necked flask, equipped with a reflux condenser and thermometer. In the reaction system, 20 mL of titanium isopropoxide (purity ≥ 99.5%, Thermo Fisher, Waltham, MA, USA) was added dropwise for 2 h at a slow stirring rate (50 rpm) at 60 °C. The development of white grains in the mixture marked the completion of the reaction. Obtained particles (bTiO_2_) were washed with ethanol (purity > 70%, Reahem, Novi Sad, Serbia) and deionized water (18 MΩ cm) before being dried for 2 h at 70 °C.

#### 4.1.2. Preparation of Y_2_O_3_-bTiO_2_

In a reaction flask, 5 g of the previously synthesized bTiO_2_ particles were dispersed in 20 mL of an aqueous solution containing 0.1 M YCl_3_ (purity > 99.5%, Fluka, Frankfurt am Main, Germany) and 0.01 M glucose (purity > 99.5%, THG plc, Manchester, UK), and the mixture was vigorously stirred at 500 rpm. Subsequently, 5 mL of xylene (purity > 98%, Sigma Aldrich, Saint Louis, MO, USA) was added to promote the formation of a coarse emulsion, and stirring was continued under the same conditions. A solution of ammonium oxalate (purity > 99%, Thermo Fisher Scientific, Waltham, MA, USA) was then added dropwise to the suspension to facilitate the precipitation of yttrium oxalate onto the bTiO_2_ surface. The resulting composite was allowed to age for 60 min in the dark, followed by rinsing with ethanol and then distilled water, and dried for 12 h at 70 °C. Finally, the material was calcined at 700 °C for 2 h in a resistive furnace, leading to the thermal decomposition of yttrium oxalate and the formation of the Y_2_O_3_–bTiO_2_ photocatalyst.

### 4.2. Structural Characterization of Fabricated Composite

The fabricated photocatalyst underwent detailed structural characterization.

The presence of functional groups on the material surface before and after modification were determined by FTIR technique (Thermo Scientific Nicolet iS50, Thermo Fisher Scientific, Waltham, MA, USA). Scans were collected in the range of 4000–400 cm^−1^.

The mineral formation of fabricated composite was depicted by X-ray diffraction (XRD, Philips PW 1710/1820, Eindhoven, The Netherlands) in order to identify the phase composition. Scans were recorded in the range of 4–65° 2θ, counting for 1 s per 0.02° step. Surface morphology after modification of base TiO_2_ was characterized by SEM (JEOL JSM-7001F, Tokyo, Japan). The EDS (Oxford Xplore 15, High Wycombe, UK) technique was coupled with SEM in order to determine distribution of elements into the photocatalyst. The SEM operated in high vacuum mode (0.1 mPa) at an accelerating voltage of 20 kV and a probe current of 10 nA.

TEM analysis (JEOL JEM-2100, Jeol Ltd., Tokyo, Japan) was performed with the aim to determine particle dimension and shape of initial and modified photocatalyst. The TEM operated at 200 keV and samples were ultrasonically dispersed and placed onto lacey, carbon-coated cupper grids (Ted Pella, Redding, CA, USA).

The XPS technique (Genesis XPS spectrometer, Ulvac-PHI, Chanhassen, MN, USA) was performed in order to determine surface interaction between base TiO_2_ and deposited Y_2_O_3_. Spectrometer is equipped with the Al-monochromatic source with energy of X-rays 1486 eV. The analyzed area was 0.1 mm in diameter, and the analyzed depth was about 3–5 nm. We used a low-energy electron and ion gun to reduce charge neutralization. High-energy resolution spectra for identification of oxidation states were acquired with pass energy of 27 eV and energy resolution of 0.7 eV. Accuracy of the binding energy is ±0.3 eV. For XPS spectra, the energy scale was aligned by C 1s peak at 285.0 eV, which is characteristic for C-C/C-H bonds in organic materials, which were expected on the surface. Quantification of surface composition was performed from XPS peak intensities, taking into account relative sensitivity factors provided by the instrument manufacturer. XPS data were processed by software Multipak ver. 9.9.1 from Physical Electronics, Ulvac-PHI (Chanhassen, MN, USA). Two places were analyzed on every sample.

Diffuse reflectance spectroscopy (DRS) was utilized to examine the bandgap energy together with the light-absorbing properties in the wavelength range of 200–800 nm. Thus, the spectra were obtained using Shimadzu UV-2600 (Shimadzu, Kyoto, Japan) and provided with an integrated sphere (ISR-2600 Plus, Shimadzu, Kyoto, Japan).

### 4.3. Photocatalytical Degradation of RB5

Photocatalytical reactions were performed according to the procedure as described in a paper from Jovanović et al. 2024 [67]. The photocatalytic experiments were conducted in a 200 mL quartz reactor containing the fabricated photocatalyst and an aqueous solution of Reactive Black 5 (Sigma-Aldrich, Saint Louis, MO, USA). The initial dye concentration was adjusted in the range of 10–30 mg/L, while the amount of photocatalyst was varied between 50 and 200 mg/L. A 300 W UV–Vis lamp (Osram Vitalux, Munich, Germany) was positioned above the reactor as the irradiation source. Prior to illumination, the suspension was stirred in the dark for 30 min to establish adsorption–desorption equilibrium, after which photocatalytic reactions were initiated under simulated sunlight irradiation.

### 4.4. Degradation Kinetics and Quantum Yield Determination

At desired times, aliquots were collected from the reactor, filtered through a 0.22 µm syringe filter, and placed into UV/Vis spectrophotometer (Shimadzu 1600, Kyoto, Japan) with the aim of determining the quality of the proposed degradation system.

Kinetics calculation of photocatalysis was performed using the Langmuir–Hinshelwood Equation [68]:

*ln*(*C*_0_/*C*) = −*k* × *t* (1)

where *C*_0_ and *C* are concentrations of RB5 at the beginning and at regular time *t* (min); *k* (min^−1^) is the reaction rate constant.

Quantum yield (*Φ*) of photodegradation reactions were determined following procedure from Jovanović et al. 2023 [69]. A prepared chemical actinometer K_3_[Fe(C_2_O_4_)_3_] was used in the measurement of absorbed photons in the reaction solution by adding the reaction system together with the pollutant solution. During irradiation of potassium ferrioxalate, Fe^3+^ reduced into Fe^2+^, a concentration which was used for calculating the quantum yield. *Φ* was calculated using the following Formula [70]:

(2) $$\Phi =\frac{k}{E_{\mathrm{avg}}}\times \frac{1}{2.303\times \varepsilon }$$

where *ε* is the molar absorption coefficient of RB5 (M^−1^cm^−1^), *k* is the pseudo-first-order rate coefficient (s^−1^), and *E*_avg_ is the average incident photon irradiance (J).

## 5. Conclusions

In this study, a novel Y_2_O_3_–bTiO_2_ photocatalyst was successfully fabricated by depositing yttrium oxide onto a green-synthesized, biobased TiO_2_ support. The composite exhibited enhanced structural and morphological characteristics compared to the pristine bTiO_2_, including sharper particle edges and improved crystallinity as a result of thermal activation at 700 °C. FTIR, XPS, and SEM-EDX analyses confirmed the effective incorporation of 8.5 mass% Y_2_O_3_ uniformly distributed over the TiO_2_ surface, ensuring strong interfacial bonding and stability of the heterostructure.

The photocatalytic activity of the Y_2_O_3_–bTiO_2_ material was evaluated through the degradation of Reactive Black 5 (RB5) under simulated sunlight irradiation. Nearly complete removal of RB5 was achieved within 60 min at room temperature, with optimal performance observed using 10 mg L^−1^ dye concentration and 0.20 g/L photocatalyst dosage. The process followed pseudo-first-order kinetics, and the obtained rate constants (*k*) as well as quantum yield values were consistent with the superior photocatalytic efficiency of the composite. Although higher dye concentrations led to reduced degradation rates due to hindered light penetration and competition for active sites, the Y_2_O_3_–bTiO_2_ system remained effective across all tested conditions.

Reusability tests demonstrated strong durability of the composite, retaining 82–85% of its initial efficiency after five consecutive cycles, with no detectable yttrium leaching. The maintained performance indicates minimal structural deterioration and confirms the composite’s potential for practical application.

Overall, the results highlight that Y_2_O_3_ modification significantly improves the photocatalytic capability of green-synthesized TiO_2_ without a significant increase in material complexity or production cost. Therefore, the Y_2_O_3_–bTiO_2_ photocatalyst represents a promising and sustainable candidate for advanced wastewater treatment—particularly for effluents containing azo dyes such as RB5. Future work should focus on applying this catalyst in real wastewater matrices, assessing the influence of coexisting ions and organic species, and expanding the pollutant scope to include pharmaceuticals and agricultural fungicides commonly detected in aqueous environments.

## Figures and Tables

**Table 1:** Presence of elements in two materials, in at.%.

Material	C	O	Ti	Cl	Y
bTiO_2_	29	53.3	17.7	0	0
Y_2_O_3_-bTiO_2_	33.7	45.7	12.7	4.9	3

**Table 2:** Summarized PFO rate constants of RB5 photodegradation.

C_0_(RB5) (mg/L)	k ± SD * (min^−1^)	t_0.5_ (min)	R^2^
10	0.064 ± 0.0058	10.9	0.98
15	0.038 ± 0.0041	13.1	0.95
20	0.030 ± 0.0029	15.9	0.95
25	0.019 ± 0.0016	34.6	0.95
30	0.015 ± 0.00035	46.2	0.99

* SD—standard deviation.

**Table 3:** Calculated values of quantum yield, *Φ*, for photocatalytic reaction.

C_0_(RB5) (mg/L)	*Φ*
10	0.61
15	0.36
20	0.27
25	0.16
30	0.11

**Table 4:** Short comparative analysis of photocatalytic degradation parameters of various organic pollutants.

Pollutant	C_0_ (mg/L)	Catalyst	k (min^−1^)	Degradation Time (min)	Efficiency (%)	Catalyst Amount (mg/L)	Ref.
Phenol	19.75	Y-TiO_2_	0.0026	-	14	5	[12]
Methyl orange	10	Y-TiO_2_-H_2_	0.1746	80	99	100	[14]
Reactive Black 5	20	Y_2_O_3_/TiO_2_-Loaded Polyester Fabric	0.47846	150	83	-	[15]
Methyl orange	20	Ag-TiO_2_/Y_2_O_3_	0.00984	180	80.3	1000	[18]
Methyl orange	25	Y_2_O_3_/TiO_2_-Y_2_TiO_5_/CNTs	-	90	90	-	[20]
Reactive Black 5	10	Y_2_O_3_/bTiO_2_	0.064	90	99	200	Our study

## Data Availability

The data presented in this study are available on request from the corresponding author.

## Abbreviations

The following abbreviations are used in this manuscript:

RB5	Reactive Black 5
AOPs	Advanced Oxidation Processes
PFO	Pseudo-First-Order
FTIR	Fourier Transform Infrared Spectroscopy
SEM	Scanning Electron Microscopy
EDX	Energy-Dispersive X-Ray
XPS	X-ray Photoelectron Spectroscopy
UV/Vis	Ultraviolet–Visible Spectroscopy
